# Cancer genomics predicts disease relapse and therapeutic response to neoadjuvant chemotherapy of hormone sensitive breast cancers

**DOI:** 10.1038/s41598-020-65055-4

**Published:** 2020-05-18

**Authors:** Jieqiang Zhu, Levan Muskhelishvili, Weida Tong, Jürgen Borlak, Minjun Chen

**Affiliations:** 1 Division of Bioinformatics and Biostatistics, National Center for Toxicological Research, U.S. Food and Drug Administration, Jefferson, Arkansas 72079 USA; 2 Toxicologic Pathology Associates, National Center for Toxicological Research, U.S. Food and Drug Administration, Jefferson, Arkansas 72079 USA; 3 Center of Pharmacology and Toxicology, Hannover Medical School, Hannover, Germany

**Keywords:** Breast cancer, Cancer genomics

## Abstract

Several studies provide insight into the landscape of breast cancer genomics with the genomic characterization of tumors offering exceptional opportunities in defining therapies tailored to the patient’s specific need. However, translating genomic data into personalized treatment regimens has been hampered partly due to uncertainties in deviating from guideline based clinical protocols. Here we report a genomic approach to predict favorable outcome to treatment responses thus enabling personalized medicine in the selection of specific treatment regimens. The genomic data were divided into a training set of N = 835 cases and a validation set consisting of 1315 hormone sensitive, 634 triple negative breast cancer (TNBC) and 1365 breast cancer patients with information on neoadjuvant chemotherapy responses. Patients were selected by the following criteria: estrogen receptor (ER) status, lymph node invasion, recurrence free survival. The k-means classification algorithm delineated clusters with low- and high- expression of genes related to recurrence of disease; a multivariate Cox’s proportional hazard model defined recurrence risk for disease. Classifier genes were validated by Immunohistochemistry (IHC) using tissue microarray sections containing both normal and cancerous tissues and by evaluating findings deposited in the human protein atlas repository. Based on the leave-on-out cross validation procedure of 4 independent data sets we identified 51-genes associated with disease relapse and selected 10, i.e. *TOP2A, AURKA, CKS2, CCNB2, CDK1* SLC19A1, *E2F8, E2F1, PRC1, KIF11* for in depth validation. Expression of the mechanistically linked disease regulated genes significantly correlated with recurrence free survival among ER-positive and triple negative breast cancer patients and was independent of age, tumor size, histological grade and node status. Importantly, the classifier genes predicted pathological complete responses to neoadjuvant chemotherapy (P < 0.001) with high expression of these genes being associated with an improved therapeutic response toward two different anthracycline-taxane regimens; thus, highlighting the prospective for precision medicine. Our study demonstrates the potential of classifier genes to predict risk for disease relapse and treatment response to chemotherapies. The classifier genes enable rational selection of patients who benefit best from a given chemotherapy thus providing the best possible care. The findings encourage independent clinical validation.

## Introduction

Breast cancer is the top-ranking malignancy in females and accounts for about 25% of all cancers among women. Except for triple negative breast cancer, the 5-year survival is typically >90%. Despite advances in early detection and therefore improved outcomes an approximately 42,260 breast cancer deaths are expected for the US in 2019^1^. Next to surgery chemotherapy is an important treatment option and was shown to decrease the mortality rates of breast cancers significantly^2^. In the meantime, chemotherapy is associated with significant toxicity; nevertheless, is based on the paradigm of maximum tolerated doses (MTD)^2,3^. About 60% of breast cancer patients receive chemotherapy, and the decision to treat is justified based on clinical and histological features^4^; even though, patients may not derive sufficient benefit after being given toxic chemotherapeutics^2^. Thus, identifying patients who would benefit from a given chemotherapy is a prior task^5,6^, and recent studies imply that genetic screening can be a promising tool to enable personalized chemotherapy regimens of cancer patients^7^.

A number of testing systems have been considered useful to predict prognostic outcome in hormone sensitive breast cancers^8^ and are recommended by the American Society of Clinical Oncology(ASCO), and National Comprehensive Cancer Network guidelines^9,10^. However, despite the significant advances in the field, limitations still exist. First, none of the testing systems have demonstrated the capability to predict treatment responses and therefore do not assist in the selection of specific treatment regimens^9^. Second, most testing systems inform on cell proliferation events^11^, while other mechanistic and therapeutically relevant information are not fully considered, especially information on the modulation of drug targets for breast cancer therapies.

Importantly, cell cycle genes play an essential role in cancer development and certain gene products, such as polo-like kinas 1(PLK1) and Aurora Kinases, are over-expressed in human cancers and are therefore attractive drug targets for cancer therapy. The cyclin-dependent kinase CDK4/6 inhibitors, i.e. palbociclib, ribociclib and abemaciclib, have been approved by the US FDA for the treatment of advanced stages of ER positive breast cancers^12^ while other cell cycle proteins are considered novel drug targets and are under clinical evaluation^13^.

To assess the effectiveness of a given chemotherapy the so called pathologic complete response (pCR) is considered. pCR is defined by the complete lack of signs for cancer relapse after radiation and/or chemotherapy. However, predicting pCR based on genetic screening has not been attempted nor is it certain that pCR serves as a surrogate endpoint for improved overall survival^14^. Therefore, our study aimed to develop a gene expression signature that can be used to identify patients with improved response to a given chemotherapy. As a proof-of-concept for genome guided chemotherapy, we entrained signature genes as predictors to identify high-risk breast cancers and to select responders for chemotherapy. We performed a discovery analysis on 835 ER-positive breast cancers and identified 10 genes which were associated with recurrence risk of breast cancers. We then designed an algorithm to calculate the average expression of these genes as means to quantify risk of recurrence. The predictive performance of the classifier genes was independently evaluated among high-risk patients using large congregated datasets including 1315 ER-positive breast cancers and 634 triple negative breast cancer patients (TNBC). Finally, the proposed gene signature was evaluated for their sensitivities to neoadjuvant chemotherapy by considering 1365 breast cancers, most of which are hormone sensitive.

## Materials and methods

### Datasets

The training cohort (n = 835) comprised four data sets (i.e. GSE4922^15^, GSE17705^16^, GSE7390^17^, GSE2034^18^) which were selected by the following criteria: ER-receptor status, lymph node invasion, recurrence free survival data, a minimal number of patients, i.e. >100, microarray data generated on the same platform (Affymetrix, Inc., Santa Clara, CA, USA), and results were published in a quality peer-reviewed journal. All data were retrieved from the NCBI Gene Expression Omnibus (GEO, https://www.ncbi.nlm.nih.gov/geo/).

The validation cohort consisted of 1315 hormone sensitive breast cancers^19^, 634 triple negative breast cancers, and 1365 breast cancers with neoadjuvant chemotherapy responses (Table 1). The data sources for each cohort are detailed in Supplemental Table S1.

**Table 1:** Patient characteristics of the breast cancer study cohorts.

Characteristics	Training cohort	ER positive validation cohort	TNBC cohort	Neoadjuvant chemotherapy cohort
	(n = 835)	(n = 1315)	(n = 634)	(n = 1365)
**Age, years**				
<=50	142(17.0%)	399(30.3%)	219(34.5%)	668(48.9%)
>50	197(23.6%)	811(61.7%)	312(49.2%)	574(42.1%)
Unknown	496(59.4%)	105(8.0%)	103(16.2%)	123(9.0%)
**Tumor size, mm**				
<20	188(22.5%)	302(23.0%)	73(11.5%)	93(6.8%)
20–50	146(17.5%)	638(48.5%)	200(31.5%)	537(39.3%)
>50	5(0.6%)	219(16.7%)	138(21.8%)	507(37.1%)
Unknown	496(59.4%)	156(11.9%)	223(35.2%)	228(16.7%)
**Lymph node status**				
N1	0(0.0%)	174(13.2%)	85(13.4%)	541(39.6%)
N2	0(0.0%)	49(3.7%)	29(4.6%)	142(10.4%)
N3	0(0.0%)	31(2.4%)	22(3.5%)	95(7.0%)
Positive but unspecified	181(21.7%)	455(34.6%)	112(17.7%)	3(0.2%)
Negative	654(78.3%)	606(46.1%)	241(38.0%)	363(26.6%)
Unknown	0(0.0%)	0(0.0%)	145(22.9%)	221(16.2%)
**Histological grade**				
Well differentiated	91(10.9%)	227(17.3%)	16(2.5%)	61(4.5%)
Moderately differentiated	179(21.4%)	633(48.1%)	67(10.6%)	385(28.2%)
Poorly differentiated	67(8.0%)	368(28.0%)	375(59.1%)	611(44.8%)
Unknown	498(59.6%)	87(6.6%)	176(27.8%)	285(20.9%)
**Hormone status**				
ER + & PR +	0(0.0%)	839(63.8%)	0(0.0%)	448(32.8%)
ER + & PR−	0(0.0%)	210(16.0%)	0(0.0%)	154(11.3%)
ER- & PR +	0(0.0%)	0(0.0%)	0(0.0%)	43(32.0%)
ER− & PR−	0(0.0%)	0(0.0%)	634(100.0%)	432(31.6%)
Unknown	835(100.0%)	266(20.2%)	0(0.0%)	288(21.1%)
**EGFR/HER-2 status**				
Overexpression	0(0.0%)	87(6.6%)	0(0.0%)	262(19.2%)
Negative	0(0.0%)	745(56.7%)	634(100.0%)	891(65.3%)
Unknown	835(100.0%)	483(36.7%)	0(0.0%)	212(15.5%)
**Chemotherapy response**				
Pathological complete response	NA	30(2.3%)	57(9.0%)	334(24.5%)
Residual disease	NA	255(19.4%)	113(17.8%)	1031(75.5%)
Unknown	NA	1030(78.3%)	464(73.2%)	0(0.0%)
**Years of follow-up**				
Median (range)	8.3(0.0–19.1)	5.1(0.0–17.8)	3.1(0.0–15.8)	2.7(0.1–7.4)

In addition, we considered the genomic data set GSE15852 with information on paired cancerous and normal tissues to confirm the discrimination power of the classifier genes.

### Identification of the classifier genes

**Figure 1:**
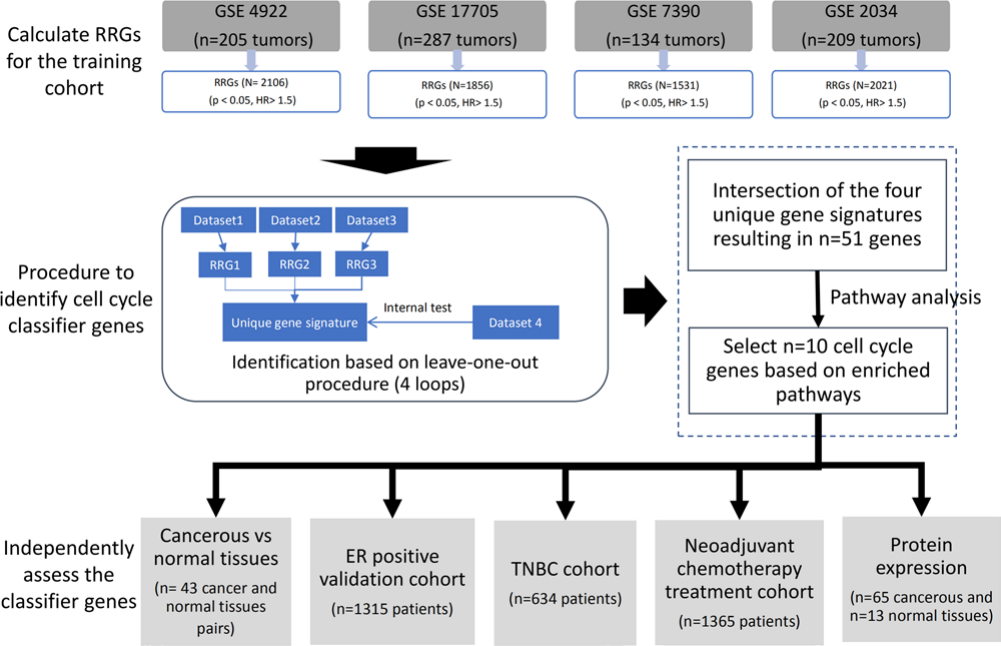
Study workflow of the development of the classifier gene signature by incorporating biological pathway knowledge into the bioinformatics process. RRG: relapse related gene.

Figure 1 depicts the overall study workflow with three separated tiers: (1) to identify recurrence of related genes (RRGs) for breast cancer; (2) define enriched pathway and entrain a gene signature predictive for RRGs; (3) validate the gene signature among ER-positive and triple negative breast cancers for treatment response to chemotherapies.

The GEO data sets GSE4922, GSE17705, GSE7390 and GSE2034, were retrieved from the public repository and analyzed for RRGs. Next, a k-means (k = 2) classification algorithm^20^ was applied to individual gene expression data. This revealed clusters of breast cancers with low- and high- gene expression data. Then, a Cox’s proportional hazard model was developed to correlate low- and high- gene expression data with the patient’s recurrence risk for disease and a hazard ratio and p-value for each gene was calculated. Genes with a hazard ratio (>1.5) and a significant p-value (<0.05) were defined as RRG.

Subsequently, a leave-one-out procedure was computed to cross-validate the predictive power of the classifier genes. In each run, one dataset is left out (e.g. GSE4922), and the other datasets (e.g. GSE 17705, GSE7390, GSE2034) will generate three RRG lists separately. Only the common genes in these three lists were left and applied to the left-out dataset (e.g. GSE4922) to assess its correlation with recurrence. The leave-one-out procedure was repeated with all datasets.

### Pathway analysis of the classifier genes

The selected gene list was imported into the Ingenuity Pathway Analysis (IPA) software, i.e. an online database widely used, to identify the statistically enriched functional pathways. P < 0.05 was considered as statistically significance.

### An algorithm to calculate the expression of the classifier genes

A simple algorithm was designed to calculate the average expression intensity of the selected genes, which will be used to quantify the risk grade of breast cancers.

$${\rm{Average}}\,{\rm{expression}}={\sum }_{({\rm{i}}=1)}^{{\rm{N}}}{\rm{Intensity}}({\rm{i}})/{\rm{N}}$$

in which Intensity(i) represents the expression level of gene i and N is the total number of the selected genes. The intensity of gene expression is based on log2 at the range of 4 to 14, with a median value of about 8.5; therefore, we categorized breast cancers into low expression (<7.5), intermediate expression (7.5–9) and high expression group (>9) based on the average expression of the classifier genes.

### Performance of the classifier genes with other multigene signatures

We draw comparison between the newly developed signature genes and the multigene signatures recommended by the ASCO guideline^9^, i.e. PAM50 (n= 50 genes)^21,22^, Oncotype DX (n= 16 genes by excluding 5 reference genes)^23^, Breast Cancer Index (BCI, n= 7 genes)^24,25^. Note, MammaPrint^26,27^ was not included in the comparison since of the original 70 predictor genes only 53 genes are disclosed. For a consistent comparison, a k-means method was used to categorize patients into low- and high- expression groups. Agreement charts for two-by-two gene signature comparisons between their low- and high- expression groups assignments were conducted.

### Immunohistochemical analysis of tissue microarray

Tissue microarray (TMA) sections containing both normal and cancerous breast tissues were retrieved from Cooperative Human Tissue Network, the University of Virginia. TMAs were deparaffinized and rehydrated using standard methods. The sections were then sequentially incubated with rabbit polyclonal anti-CDK1 (Sigma, St. Louis, MO), biotin-conjugated goat anti-rabbit, and ExtrAvidin Peroxidase (ExtrAvidin Kit, Sigma). Staining was developed with liquid DAB substrate (DAKO, Carpinteria, CA), sections were counterstained with hematoxylin, and mounted with Permount (Fisher Scientific, Pittsburgh, PA). Stained TMAs were scanned, and digital images were obtained with Aperio Scanscope System (Leica Biosystems, Vista CA). The intensity of CDK1 staining in a tissue from each patient was evaluated with the Positive Pixel Count Algorithm (Leica Biosystems). This algorithm quantifies the amount of specific stain present in a digital slide by evaluating an average intensity of all pixels for subsequent calculation of the optical density and the proportion of positively stained area.

### Statistical analysis

All statistical analyses were conducted with the JMP software (SAS Institute Inc., version 12.1.0) or the R software (version 3.4.2) which included the Bioconductor package (version 3.5) and the R-Studio package (version 1.0.153). All tests were two-sided; a P < 0.05 was considered as statistically significance. We tested the hypothesis that the proportion of patients who are free of recurrence would be significantly higher in the low expression group (<7.5) than in the high expression group (>9). The Kaplan-Meier analysis was computed to estimate the variance of recurrence free survival over time by the Greenwood variance estimate; a P value of less than 0.05 from log-rank test was considered statistically significant. A multivariate Cox model was developed to test the recurrence free survival in relation to age, tumor size, histological grade, and status of lymph nodes with or without the consideration of the signature genes. The pathway enrichment analysis with a p-value <0.05 was considered as statistically significant. Additionally, we used the R stats base package and pca3d (version 0.10) package to display 3-D score plot of tissues distribution based on the newly developed signature genes.

## Results

### Identification and validation of classifier genes to predict disease relapse

We collected data from 835 ER-positive breast cancer patients as training cohort; a set of 51-genes associated with recurrence were obtained by intersection of four unique gene lists derived from the leave-one-out procedure depicted in Fig. 1. Importantly, of these genes at least 17 are regulated by the estrogen receptor and include the proliferation index Ki67 (Supplemental Table S2). The 51 genes were imported into the IPA database to enumerate the statistically enriched functional pathways (Table 2) and apart from the estrogen-mediated pathway, cell cycle regulation pathways are prominent. Based on the pathway enrichment analysis we selected 10 genes highlighted in Table 2 as classifier for further validation.

**Table 2:** Enriched functional pathways associated with 51 genes that were associated with recurrence-free survival (P < 0.001). The 10 selected classifier genes are highlighted in bold.

Ingenuity Canonical Pathways	P-value	Molecules
Cell Cycle: G2/M DNA Damage Checkpoint Regulation	8.71E-08	**TOP2A, AURKA, CKS2, CCNB2, CDK1**
Role of CHK Proteins in Cell Cycle Checkpoint Control	8.51E-06	**SLC19A1, E2F8, E2F1**, CDK1
Mitotic Roles of Polo-Like Kinase	1.51E-05	**PRC1**, CCNB2, **KIF11**, CDK1
Estrogen-mediated S-phase Entry	2.75E-05	E2F8,E2F1,CDK1
Cyclins and Cell Cycle Regulation	3.47E-05	E2F8,E2F1,CCNB2,CDK1
Protein Ubiquitination Pathway	0.0004	HSPB1,UBE2S,PSMD7,UBE2C,PSMD2
Role of BRCA1 in DNA Damage Response	0.0008	SLC19A1,E2F8,E2F1
DNA damage-induced 14–3–3σ Signaling	0.0009	CCNB2,CDK1

**Figure 2:**
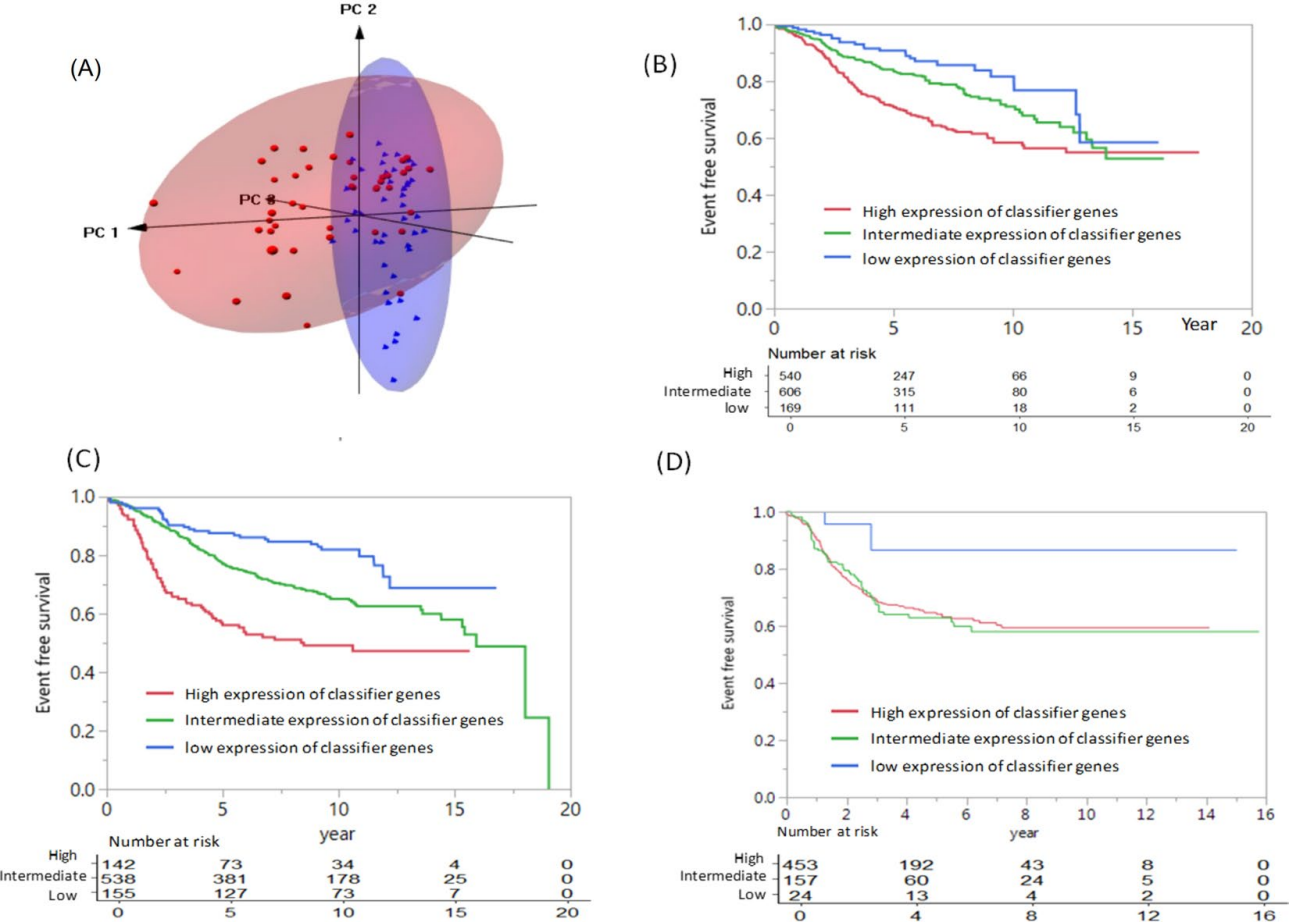
(**A**) The 3D PCA score plot of the classifier gene signature for n = 43 patients of paired cancer and normal breast tissues retrieved from GEO dataset of GSE15852. The red and blue dots represent the cancer and normal breast tissues, respectively; (**B**) Kaplan–Meier event-free survival analysis for the classifier genes in the validation cohort of n = 1315 ER-positive breast cancers (P < 0.001); (**C**) Kaplan–Meier event-free survival analysis for the classifier genes in the training cohort of n = 835 ER-positive breast cancers (P < 0.001); (**D**) Kaplan-Meier analysis for event-free survival among TNBC patients categorized by the classifier genes. There were 3 recurrence events of 24 TNBC patients in the low genomic score group,54 recurrences of 157 TNBC patients in the intermediate group, and 101 recurrence events of 453 TNBC patients in the high score group. The difference of recurrences among the high and low expression groups is statistically significant (P=0.037).

We assessed the performance of the classifier genes for its capability to differentiate cancerous and normal breast tissues. Figure 2A presents a 3D-PCA score plot derived from pairs of cancerous and normal tissues of 43 patients. Obviously, the normal tissues (blue dots) clustered together, most of which are located within the 95% confident ranges defined by the green sphere. Conversely, most cancerous tissues (red dots) are spread in the PCA space and are away from the normal tissue cluster. This finding suggests that the classifier genes have a different expression pattern between cancerous and normal breast tissues.

We validate the capability of the classifier genes to predict the likelihood of recurrence by using 1315 ER-positive breast cancers collected from 13 published datasets in the GEO database (Supplemental Table S1). The Kaplan-Meier survival analysis estimated 67% (95% CI: 65%-69%) patients have the 10-year recurrent-free survival after cancer diagnosis (Supplemental Table S3). Furthermore, the patients were categorized into low (<7.5), intermediate (7.5–9) and high expression (>9) groups as defined by the classifier genes. Figure 2B shows that the expression of classifier genes correlated with the grade of recurrence risk across time; specifically, the proportion of recurrence-free patients at 10 years is 82% (95% CI, 77–86%) in the low expression group, which is better than 71% (95% CI: 68–74%) in the intermediate expression group and 58% (95% CI: 55–61%) in the high expression group. The difference among the three groups is statistically significant (P < 0.001). Similar statistically significant results among high, intermediate and low expression groups were observed within the discovery cohort of 835 ER-positive cancer patients (Fig. 2C).

**Figure 3:**
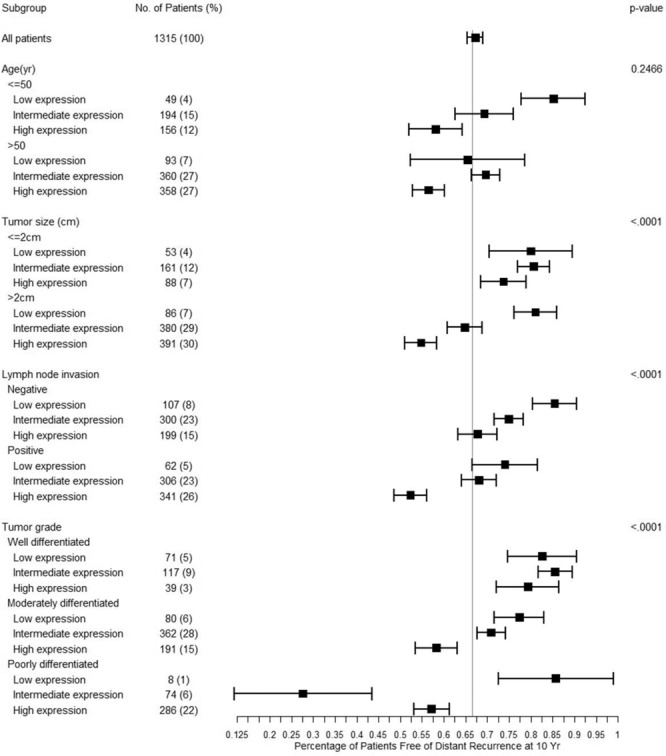
Kaplan–Meier estimates of the proportion of patients free of recurrences at 10 years, according to age, tumor size, lymph node invasion status and tumor grade.

The estimated 10-year recurrent free survival rates in the subgroups categorized by age and other clinical factors for 1315 ER-positive breast cancers are illustrated in Fig. 3. Overall, patients with a low expression of classifier genes (<7.5) have less frequent recurrent events than patients with a high expression (>9.0). Prominently, the classifier genes differentiate recurrence risk of subgroups with high risk for relapse of disease (e.g. lymph node positive, age >50 years and tumor size >2) with statistical significance (P < 0.001). For example, among 709 node positive patients 62 with low expression (<7.5) and 306 with intermediate expression (7.5–9) have 73% and 68% of the estimated 10-year recurrence-free survival rates, respectively, which is better or equal to the entire cohort (67%). In contrast, the node-positive patients with a high expression of classifier genes (>9.0) have a 10-year recurrence-free survival rate of 52%, which is statistically significantly lower than the entire cohort. For now, the expression of the classifier genes presented no or borderline statistically significance in relation to age ≤50 years, tumor size ≤ 2 cm and negative lymph node invasion. Alike, no statistically significance was obtained when considering tumor grade subgroups.

Next, a multivariate Cox model was developed to analyze the contribution of histological features of 1315 ER-positive breast cancers towards risk of recurrence (Table 3). The recurrence free survival was evaluated against age, tumor size, node status and histological grade. As expected, tumor size (P = 0.050), node status (P = 0.02), histological grade (P < 0.001) were significantly correlated with the recurrence risk of breast cancer, while age didn’t show significance (P = 0.444). In a multivariate Cox model in which recurrence free survival was estimated in relation to the classifier genes, age, tumor size, histological grade and node status, the expression of classifier genes provided significant predictive power that was independent of age, tumor size, node status and histological grade (P = 0.0035).

**Table 3:** Multivariate Cox Proportional Hazard Analysis associated with age, tumor size, lymph node invasion, histological grade, and expression of classifier genes in the validation cohort (N=1315 breast cancers).

	P Value	Hazard Ratio (95% CI)
**Analysis without expression of classifier genes**		
Age	0.4442	1.11(0.85–1.48)
Tumor size	0.0499	1.39(1.01–1.95)
Lymph node invasion	0.02	1.37(1.05–1.79)
Histological grade		
Well vs intermediate or poorly	0.0001	2.40(1.58–3.81)
Well or Intermediate vs poorly	0.1481	1.22(0.93–1.59)
Analysis with expression of the classifier genes		
Age	0.5348	1.09(0.83–1.45)
Tumor size	0.047	1.40(1.01–1.96)
Lymph node invasion	0.0314	1.34(1.03–1.75)
Histological grade		
Well vs intermediate or poorly	0.0007	2.16(1.41–3.45)
Well or Intermediate vs poorly	0.9826	1.00(0.74–1.34)
Expression of the classifier genes	0.0035	3.29(1.48–7.35)

**Figure 4:**
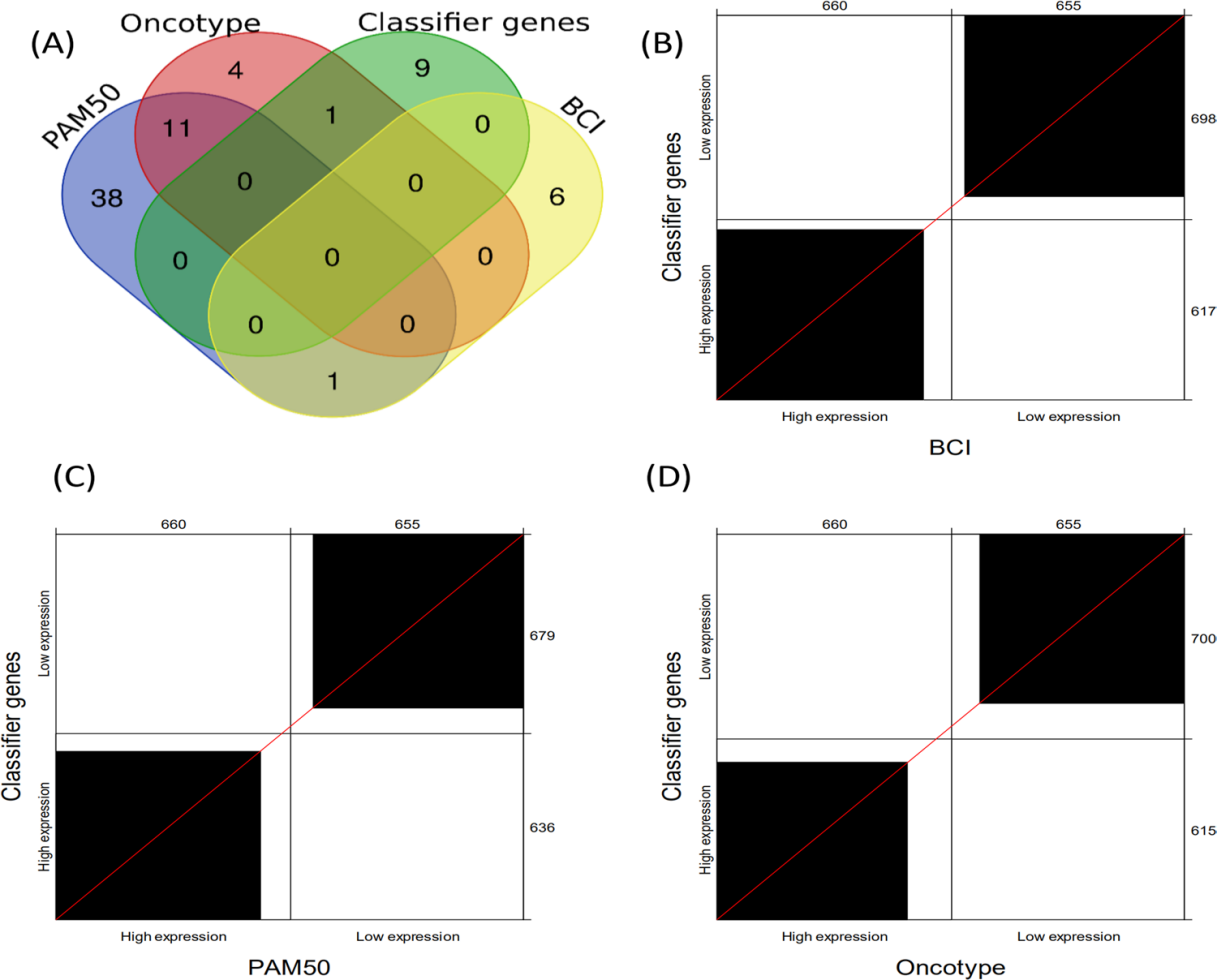
(**A**) Venn-diagram plot among different diagnostic gene signatures: the classifier genes (n = 10), PAM50 (n = 50), BCI (n = 7), and Oncotype DX (n = 16 cancer-related genes). Overall, there is no single gene in common. Agreement charts for two-by-two comparison of n = 1315 ER-positive patients in the validation cohort are displayed according to high vs low expression groups as defined by the 10 classifier genes against (**B**)-(**D**) BCI, PAM50, and Oncotype. K-means algorithm (k = 2) was used to define high versus low expression groups for all the diagnostic gene signatures.

We performed a meta-analysis and compared the classifier genes with the predictor genes defined by PAM50 (n = 50)^21,22^, Oncotype DX (n = 16), BCI (n = 7). Overall there is not a single gene in common among these diagnostic tools as illustrated in Fig. 4A. Specifically, 9 of 10 herein proposed classifier genes were not used by any of these platforms, while AURKA is shared by Oncotype. Even putting the MammaPrint into consideration, there is only PRC1 overlapping with the classifier genes. The concordance of the low- and high-expression groups defined by the classifier genes and other signatures was examined based on the 1315 validation cohort. As shown in Fig. 4(B–D), the classifier gene signature reported in the present study has good concordance with BCI, followed by PAM50 and Oncotype DX.

Of note, TNBC is a group of aggressive breast cancers with poor prognosis. So far, no gene signature was endorsed to stratify TNBC patients^9^. We collected 634 triple negative breast cancer cases to assess the prediction power of the classifier genes for recurrence risk. Specifically, we identified 3 recurrence events among 24 TNBC patients within 10 years follow-up within low expression of the classifier genes (<7.5) as compared to a 41% recurrence observed among 453 TNBC patients with high expression of the classifier genes (>9.0). The Kaplan-Meier analysis estimated a statistically significant association between expression of the classifier genes and recurrence free survival among TNBC patients (P = 0.037) (Fig. 2D).

### The classifier genes predict response to neoadjuvant chemotherapies

We evaluated whether the classifier genes can predict an individual patient’s response to neoadjuvant chemotherapy. Patients were categorized into pathological complete response (pCR) or residual diseases (RD), and the percentage of patients achieving pCR was used to determine the response rate to specific chemotherapy regimens. Data of 1365 breast cancer cases were collected from 11 datasets of GEO database (Supplemental Table S1) and included 334 pCR and 1031 RD patients. The patients achieving pCR have significant higher survival rates as compared to RD patients (Supplemental Fig. 1). The overall pCR rate is 24% without considering specific chemotherapy regimens. Importantly, the expression of the classifier genes significantly correlated with the possibility of achieving pCR after chemotherapy; i.e. the pCR rate in the low expression group is 9% (10 of 113 patients) as compared to 36% (229 of 644 patients) in the high expression group (Table 4), of which the difference is statistically significant (P < 0.001).

**Table 4:** An evaluation of the 10 mechanistically relevant classifier genes identified by pathway enrichment analysis to predict response to chemotherapy.

		The 10 classifier genes			
		pCR	RD	pCR%	P-value
All patients	All patients expression of classifier genes				
	Overall	334	1031	24%	**<0.001**
	High expression	229	415	36%	
	Intermediate expression	95	513	16%	
	Low expression	10	103	9%	
	FEC-Taxotere				
	Overall	25	41	38%	**<0.05**
	High expression	21	21	50%	
	Intermediate expression	4	20	17%	
	Low expression	0	0		
	FAC-Taxol				
	Overall	83	273	23%	**<0.001**
	High expression	57	107	35%	
	Intermediate expression	23	134	15%	
	Low expression	3	32	9%	
	Only FEC				
	Overall	30	104	22%	**<0.01**
	High expression	18	31	37%	
	Intermediate expression	12	63	16%	
	Low expression	0	10	0%	
ER +	All patients expression of classifier genes				
	Overall	92	605	13%	**<0.001**
	High expression	71	192	27%	
	Intermediate expression	18	326	5%	
	Low expression	3	87	3%	
	FEC-Taxotere				
	Overall	8	20	29%	0.1345
	High expression	6	7	46%	
	Intermediate expression	2	13	13%	
	Low expression	0	0		
	FAC-Taxol				
	Overall	20	185	10%	**<0.001**
	High expression	18	65	22%	
	Intermediate expression	1	93	1%	
	Low expression	1	27	4%	
	Only FEC				
	Overall	10	49	17%	0.4166
	High expression	5	15	25%	
	Intermediate expression	5	28	15%	
	Low expression	0	6	0%	
ER-	All patients expression of classifier genes				
	Overall	210	349	38%	**<0.001**
	High expression	129	161	44%	
	Intermediate expression	75	173	30%	
	Low expression	6	15	29%	
	FEC-Taxotere				
	Overall	17	20	46%	**<0.05**
	High expression	15	14	52%	
	Intermediate expression	2	6	25%	
	Low expression	0	0		
	FAC-Taxol				
	Overall	63	88	42%	0.1198
	High expression	39	42	48%	
	Intermediate expression	22	41	35%	
	Low expression	2	5	29%	
	Only FEC				
	Overall	20	55	27%	**<0.01**
	High expression	13	16	45%	
	Intermediate expression	7	35	17%	
	Low expression	0	4	0%	

Abbreviation: FEC, fluorouracil, epirubicin, cyclophosphamide; FAC: fluorouracil, adriamycin, cyclophosphamide.

We also assessed whether the patients with high expression of the classifier genes was associated with a higher rate of pCR among different chemotherapy regimens. As shown in Table 4 and Supplemental Table S4 eight chemotheraputic regimens were considered, i.e. only FEC (fluorouracil, epirubicin, cyclophosphamide), only FAC (fluorouracil, doxorubicin, cyclophosphamide), only Taxol, FEC + Taxotere, FAC + Taxol, FEC + Taxol+anti-HER2, FEC + Taxol, and FEC + Taxotere+ anti-HER2.The patients with high expression of the classifier genes had significantly higher pCR rates of 50% and 35% when receiving FEC + Taxotere and FAC + Taxol, respectively as compared to the overall average of 38% and 23% (P < 0.001), thus representing an improved therapeutic response rate. Another therapeutic regimen, i.e. only FEC, also showed a higher pCR (37%) with a borderline statistical significance (P < 0.05). Conversely, for the other regimens (i.e. FEC + Taxol+anti-HER2, FEC + Taxotere+ anti-HER2, FEC + Taxol, only FAC, and only Taxol) a statistically significantly higher pCR rate in the high expression group could not be established (see Supplemental Table S4).

Furthermore, we found that HER2 positive breast cancers, high histological grade and triple negative status were prone to achieve better response to chemotherapy, but not for those of low histological grade, node negative, ER and PR positive. The age, tumor size, histological phenotypes didn’t significantly affect the possibility to achieve better responses (See Supplemental Table S5).

**Figure 5:**
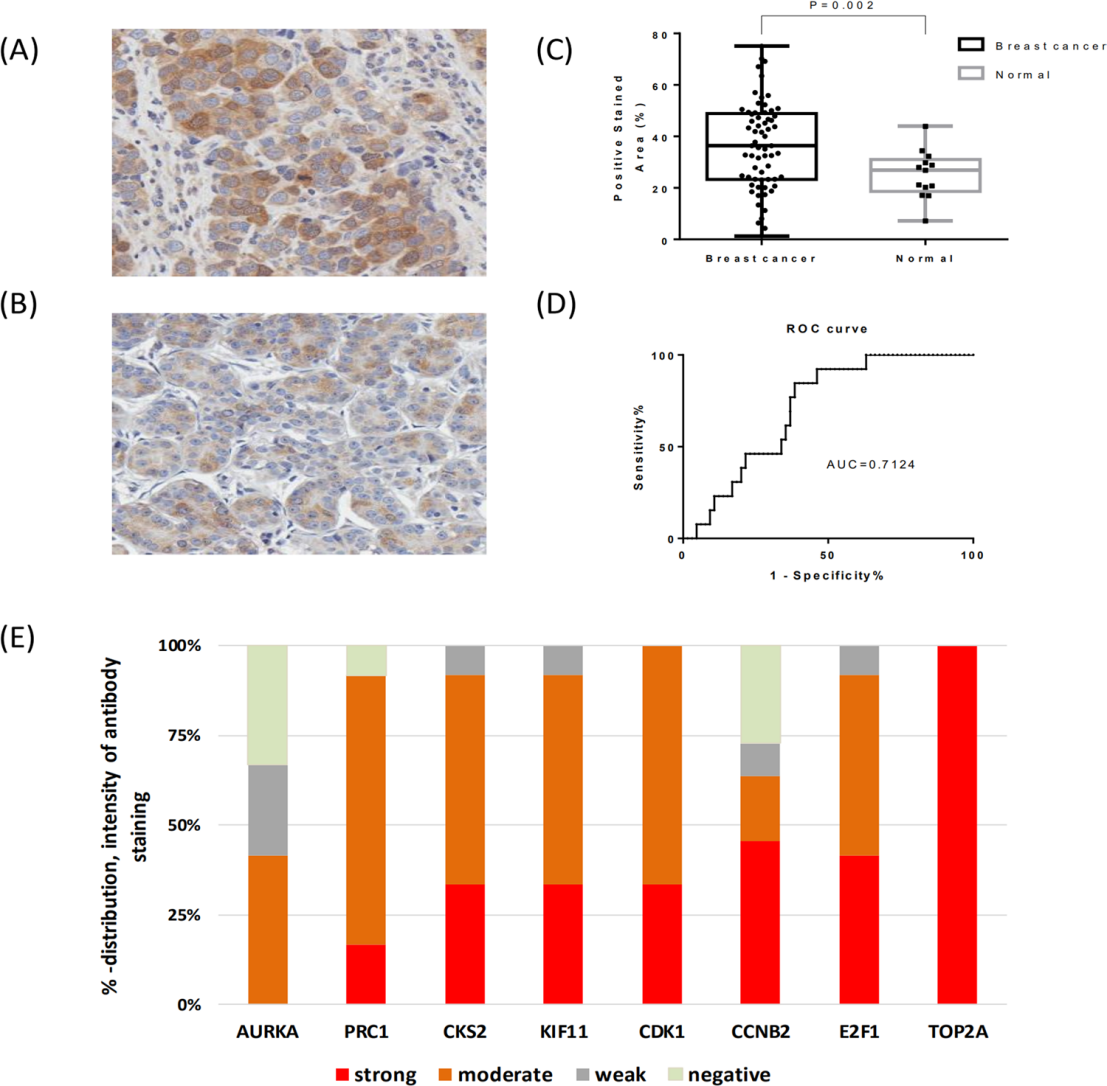
Immunohistochemical staining for CDK1 in both cancer (n = 65) and normal breast tissues (n = 13). Examples of CDK1 stained tumor (**A**) and normal breast tissue (**B,C**) boxplot highlighting percentage stained area of CDK1 in cancer and normal breast tissues. (**D**) ROC curve analysis highlighting the sensitivity and specificity for the CDK1 staining. (**E**) Immunohistochemical evaluation of expression of the classifier genes of breast cancer tissues in individual patients; data are retrieved from the human protein atlas (https://www.proteinatlas.org/).

We also examined the immunohistochemistry findings related to the 10 classifier genes. CDK1 was selected for experimental validation considering the importance of CDKs genes as therapeutic target. Immunohistochemical analysis was used to measure CDK1 protein expression among 65 breast cancers and 13 normal breast tissues. As shown in Fig. 5A–D, CDK1 expression in breast cancer tissues are statistically significantly higher than those in the normal tissues (P < 0.002) with an AUC value of 0.71. The expression of the classifier genes was also assessed by considering data deposited in the human protein atlas (https://www.proteinatlas.org/), a content-rich resource to analyze the human proteins in cells, tissues and organs. Expectedly, 8 of the 10 classifier genes were overexpressed as protein in breast cancers, including CDK1 (Fig. 5E).

## Discussion

Predicting individual responses to chemotherapy is a major breakthrough in personalized medicine and would be of great importance for therapeutic decision making. In this study, we identified 10 genes and a set of emerging targets for cancer therapies as classifier genes and designed a simple algorithm to quantify recurrence risk of breast cancers. These newly identified classifier genes have little overlap with published multigene signatures; however significantly correlate with risk of disease relapse and proved to be independent of age, tumor size, histological grade and node status. Importantly, high expression of the classifier genes was significantly associated with a better outcome and to achieve pCR after receiving certain chemotherapies, especially those consisting of anthracyclines and taxanes.

In the past, some multigene signatures, including Oncotype and MammaPrint, have been endorsed by clinical guidance to predict prognosis of ER-positive breast cancers. Although diverse genes are measured, their prognostic capability are largely due to proliferation predictors^11^. Other genes mechanistically linked to disease were sporadically claimed to predict breast cancer prognosis. For example, p27Kip1, was reported as low expressed among breast cancer cases with poor survival^28^.

As described above there was little overlap in signature genes among the different diagnostic tools even though the predictive power is similar. Concern has been voiced regarding the uniqueness of signature genes in predicting outcomes^29^. To overcome limitations in the data analysis we selected 4 large data sets for the training and 13 for the validation of the classifier genes (Supplemental Table S1). The newly developed signature is better in discriminating recurrence among patients with high risk of disease relapse. Especially, among patients of tumor size> 2 cm the high expression group had a significantly lower recurrence-free survival within 10-years than the intermediate or low expression groups (55% vs 65% or 85%); in the meantime, the difference among the three groups narrowed down (i.e. 75% vs 83% or 82%) and was not statistically significant among patients with a tumor size <2 cm. A similar tendency was observed for age. Conversely, the expression of cell cycle genes among node-positive patients was highly predictive with an estimated 10-year recurrence free survival rate of 80% and 50%, respectively, for the low and high expression group. These evidences suggest that the use of classifier genes can identify high-risk breast cancers with relapse.

So far, no multigene assay are endorsed by clinical guidance for predicting prognosis of triple negative breast cancers^30^. In our study, the classifier genes categorized most TNBC patients into intermediate or high expression groups. Only 20 of 465 TNBC were grouped as low expression and the estimated 85% recurrence-free survival rate is statistically significantly lower as compared to the intermediate or high expression groups. Note, CDK inhibitors were reported to suppress the growth of TNBC cells *in vitro* and *in vivo*, thus underpinning the role of cell cycle genes in the development of TNBC^31^.

Chemotherapy is an important treatment option and was shown to reduce one third of annual death rate regardless of tumor characteristics^2^. It is recommended for triple-negative, HER2-positive breast cancers and high-risk ER-positive tumors. Apart from significant toxicity patients do not necessarily benefit from chemotherapy and there are no validated predictive markers to allow the tailoring of chemotherapy regimens to individual patients^30^. Achieving pCR is associated with favorable outcomes^32^ though its predictive power as surrogate endpoint for improved overall survival has been challenged^14,33^. Notwithstanding, pCR is a valuable endpoint in assessing therapeutic response to treatment^14^. Although the overall response to chemotherapy or pCR was relatively low, i.e. 24% (Table 4); the pCR rate reported in pooled analyses of 5,000–10,000 patients is around 30% or less^14,33^. As observed in our study and by others^14^, ER-negative and HER2-positive, triple negatives and high grade tumors are more responsive to neoadjuvant chemotherapy than ER-positive, HER2-negative tumors, and the lobular subtype (Supplemental Table S5). Since the patients with a high expression of classifier genes were also at a high risk of disease relapse, it is reasonable to observe that this group achieved a higher pCR rate than those with low classifier genes expression.

Defining the optimal chemotherapy for a patient is a challenging issue^30^. Improving pCR responses could be achieved by identifying responders for specific chemotherapies, which was classified as one of priority issues for breast cancer research^30^. Patients respond differently to various regimens; for example, addition of taxanes into anchroacyclines regimens is well known to improve the efficacy of chemotherapy^34^ and even certain subgroups in triple-negative breast cancer could achieve a higher pCR rate when treated by certain regimens^35^. However, no validated biomarker was accepted to guide drug selection for chemotherapy in clinical practice^30^. In our study, patients with high expression of the classifier genes achieved a significantly higher pCR rate when treated by FEC + Taxotere and FAC + Taxol as compared to other regimens, suggesting the classifier genes can help guide the selection of patients for specific therapeutic regimens. In fact, our data (Table 4) is suggestive for an improved outcome for up to one half of patients with high expression of the classifier genes as compared with the overall treatment group.

Adding target therapy into conventional chemotherapy has shown a higher possibility of achieving clinical benefit^30^. Several CDK4/6 inhibitors (i.e. palbociclib, ribociclib and abemaciclib) were approved as first line therapy for advanced ER-positive breast cancers, and preliminary studies show CDK4/6 inhibitors with aromatase inhibitors increased therapy response rates^36^. Shown in Supplemental Table S6 are certain cell cycle genes that have been used as drug targets for developing new cancer therapies, and some therapies have been approved or are in clinical trial. Although not validated yet, it is reasonable to speculate that patients with high expression of the classifier genes could benefit from the combined chemotherapy and target therapies of CDK4/6. Furthermore, the mechanistic relevant classifier genes we identified by pathway analysis outperforms the differentially expressed genes identified by statistical machine learning method or other statistical procedures for predicting response to chemotherapy and disease relapse (see Supplemental Materials).

We wish to address some caveats. First, this is a retrospective study and the performance of classifier gene signature still needs to be validated in prospective studies. Second, the datasets used are collected from different labs without following a consistent protocol, and therefore the data quality might vary. Third, we didn’t use the original algorithm to measure the predictor genes of Oncotype and others, and therefore the predictions from these gene signatures might be not fully consistent with the original assays. Finally, a guideline for the selection of chemotherapeutic agents based on molecular profiling is still lacking; however, is needed for the development of personalized medicine.

## Conclusion

Our study demonstrates the successful identification of classifier genes to predict disease relapse and treatment response to specific chemotherapies. Thus, patients can be selected who benefit best from a specific chemotherapy.

## Supplementary information

Supplementary information.

## Data Availability

All raw are available through NCBI Gene Expression Omnibus (GEO, https://www.ncbi.nlm.nih.gov/geo/) as detailed in Supplemental Table S1.
