# Supplementary material for: Cancer genomics predicts disease relapse and therapeutic response to neoadjuvant chemotherapy of hormone sensitive breast cancers
Source: Sci Rep. 2020 May 18;10:8188. doi: 10.1038/s41598-020-65055-4 (PMC7235228; doi:10.1038/s41598-020-65055-4)
Supplement: Supplementary file 1 — Supplementary information. [file 41598_2020_65055_MOESM1_ESM.docx]

**Cancer genomics predicts disease relapse and therapeutic response to neoadjuvant chemotherapy of hormone sensitive breast cancers**

Jieqiang Zhu^1^, Levan Muskhelishvili^2^, Weida Tong^1^, Jürgen Borlak^3*^, Minjun Chen^1*^

^1^Division of Bioinformatics and Biostatistics, National Center for Toxicological Research, U.S. Food and Drug Administration, Jefferson, Arkansas 72079, USA.

^2^Toxicologic Pathology Associates, National Center for Toxicological Research, U.S. Food and Drug Administration, Jefferson, Arkansas 72079, USA.

^3^Center of Pharmacology and Toxicology, Hannover Medical School, Hannover, Germany

We compared the performance of the mechanistically relevant classifier genes with those obtained by conventional machine learning methods. Therefore, two conventional machine learning methods were used to identify differentially expressed genes (DEGs) and evaluate their predictions for disease relapse and response to neoadjuvant chemotherapy.

The analysis workflow for one of the conventional machine learning method is shown in Supplemental Figure 2A. This method is based on ordinary microarray statistical analysis to identify DEGS. Specifically, the genomic data set GSE15852 with information on 43 paired cancerous and normal tissues were used to define cancer related DEGs. A student t-test was performed to calculate statistical significance, and a cutoff for p-value < 0.00001 was set to select the top 10 ranking differential expression genes (see Supplemental Table 9).

The workflow for the second machine learning method is given in Supplemental Figure 3A. Here, K-means and Cox regression analysis was used to identify disease relapse related DEGs. The datasets GSE4992, GSE17705, GSE7390 and GSE2034 were retrieved and N=268 patients with disease relapsed within 2 years and N=84 patients without disease relapse for > 10 years were compared. Relapse related DEGs were derived as detailed in the method section. Each gene’s performance was evaluated by a Cox’s proportional hazard model and a student t-test. P-values from log rank test and the student t-test were computed to select the top 10 ranking disease relapse related genes (see Supplemental Table 10).

We also performed a Cox proportional hazard analysis for the 10 individual mechanistic relevant classifier genes for their utility in predicting disease relapse among N=1315 patients (validation set). As shown in Supplemental Table 7 and except for CKS2 with borderline significance (P=0.08) these classifier genes are significantly associated with disease relapse (P < 0.05). We also evaluated individual classifier genes for their ability to predict response to neoadjuvant chemotherapy among the treatment responsive cohort (N= 1365 patients). As shown in the Supplemental Table 8, all 10 classifier genes are statistically significant (P <0.05) based on the Chi- squares analysis.

We next compared the data to the top 10 ranking DEGs defined by conventional machine learning techniques (Supplemental Table 9,10). None of top-ranking DEGs overlapped with the classifier genes. The genes defined by microarray statistical analysis didn’t show prediction for disease relapse in the validation set of N=1315 patients (Supplemental Figure 2C) and response to chemotherapy for specific regimens (Supplemental Table 11). In the meantime, the expression of the 10 top-ranking genes defined by conventional machine learning was evaluated for statistics significance in predicting disease relapse (Supplemental Figure 3C) and response to chemotherapy (Supplemental Table 11). Importantly, the classifier genes were superior to the top-ranking genes identified by machine learning approaches as evidenced by statistical analysis for the prediction of response to chemotherapy (Supplemental Table 11). Thus, more ER positive breast cancers patients with high expression of the classifier genes benefitted from a FAC-Taxol regimen, i.e. a higher portion of pCR patients were observed, and a similar improved pCR was observed for ER negatives patients on the FEC-Taxotere and only FEC treatment regimes.

Supplemental Figure 1. Kaplan–Meier survival analysis for the patients defined by pathological complete response (pCR) or residual diseases (RD) within the treatment responsive cohort of N= 982 patients with survival data (P <0.001).


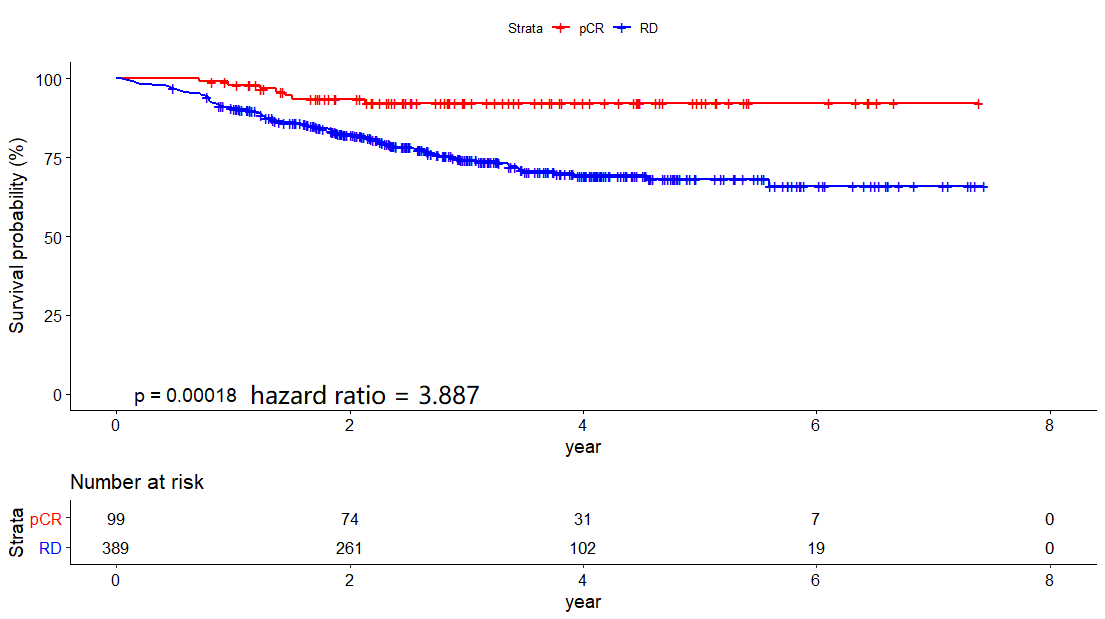


Supplemental Figure 2. Data analysis of the 10 top ranking genes defined by statistical analysis for microarray data. (A)The analysis flowchart. (B) Kaplan–Meier survival analysis of breast cancer recurrence for the training set (N=835 patients) and (C) for the validation set (N=1315 patients).

(A)


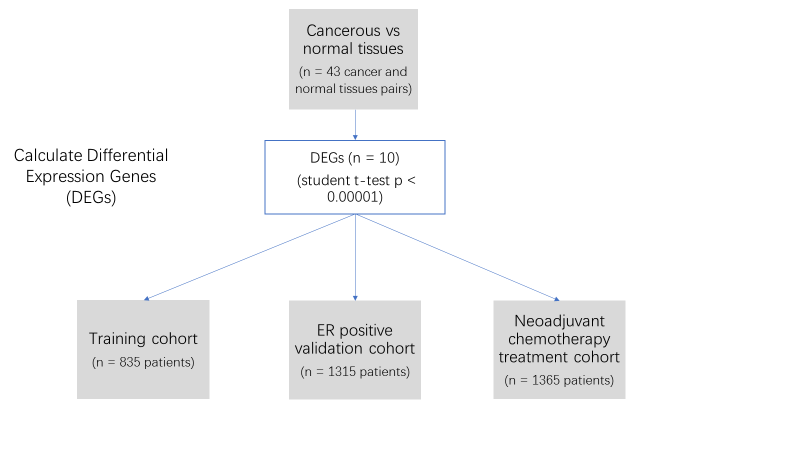


(B)


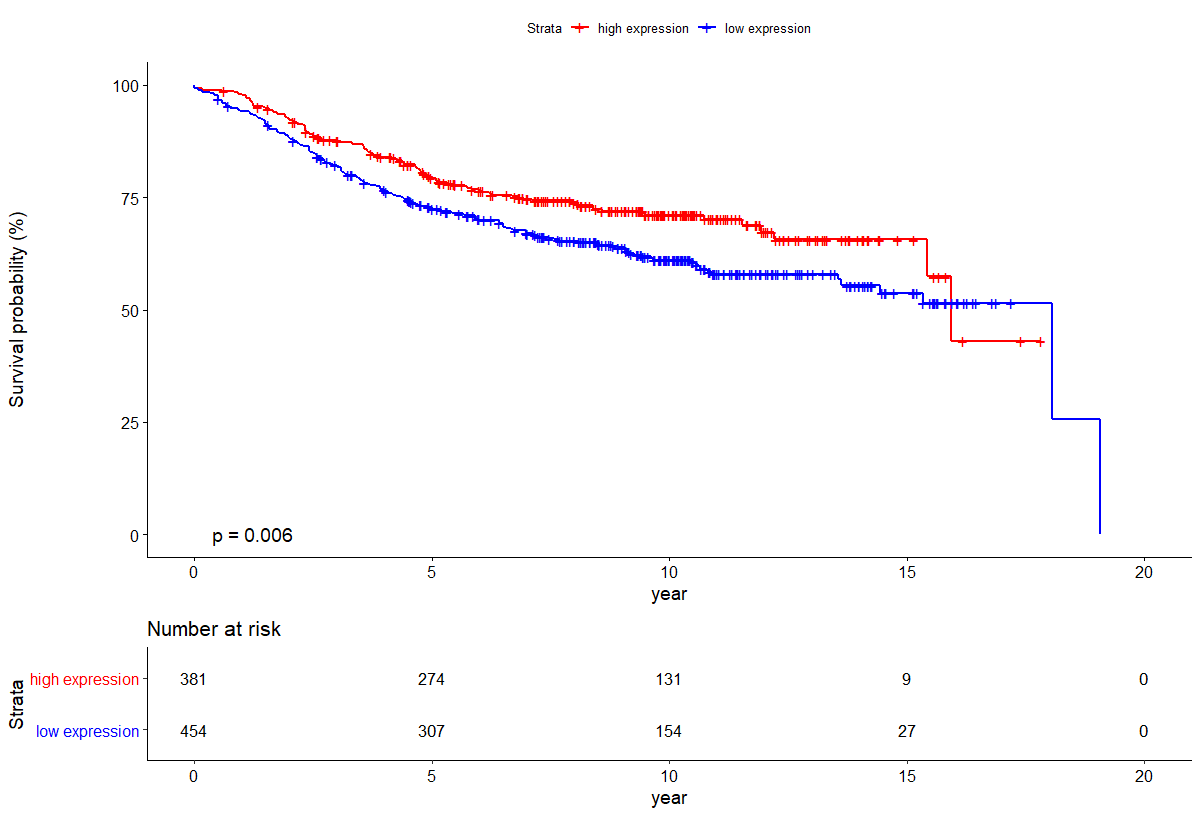


P = 0.006

(C)


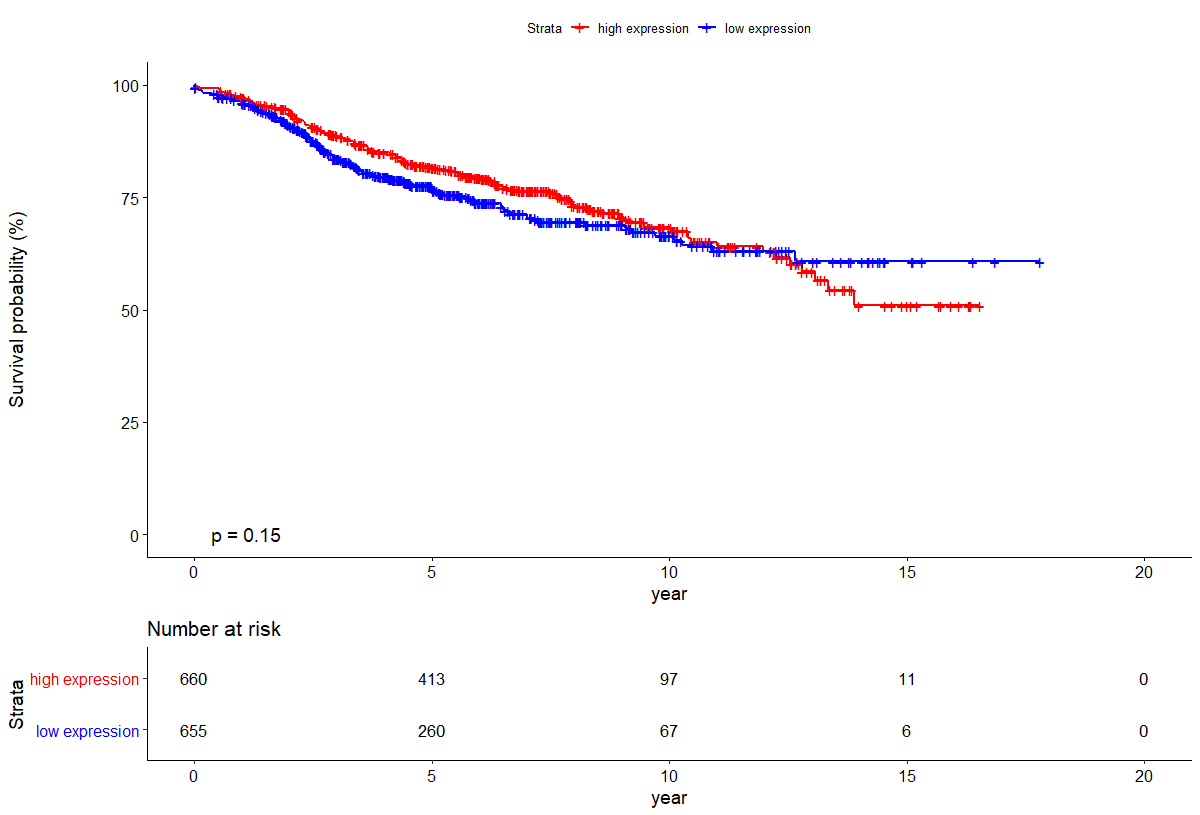


P = 0.15

(B) P = 0.15 become bigger

Supplemental Figure 3 Data analysis for the 10 top ranking genes defined by machine learning technique. (A)The analysis flowchart. (B) Kaplan–Meier survival analysis for the training set (N=835 patients) and (C) for the validation set (N=1315 patients).

(A)

(B)


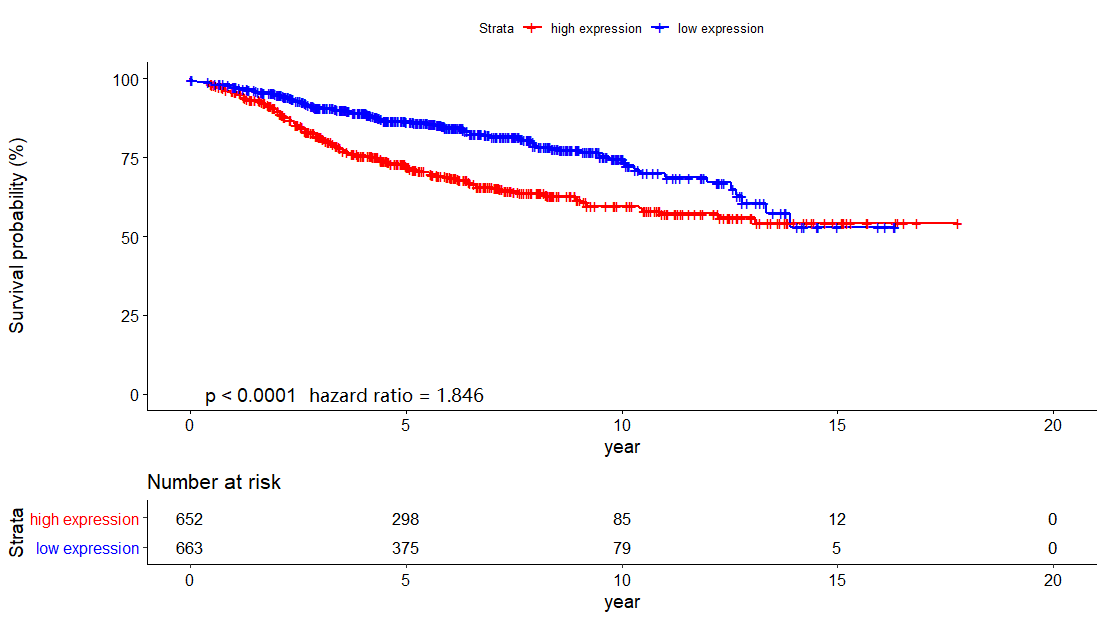


(C)


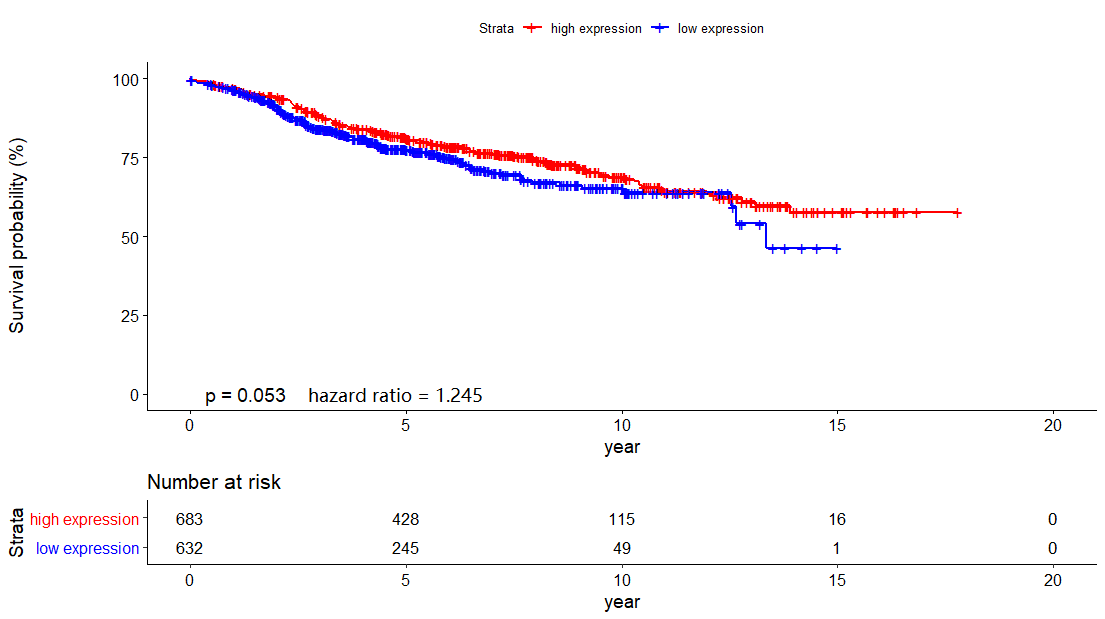


Supplemental Table S1: Microarray datasets used.

| Study cohorts | GSE datasets |
| --- | --- |
| Training cohort | GSE2034, GSE4922, GSE7390, GSE17705 |
| Validation cohort | GSE19615, GSE17907, GSE20711, GSE21653, GSE25055, GSE25065, GSE31448, GSE42568, GSE45255, GSE61304, GSE6532, GSE88770, GSE9195 |
| TNBC cohort | GSE19615, GSE17907, GSE1456, GSE20711, GSE21653, GSE25055, GSE25065, GSE31448, GSE31519, GSE45255, GSE48390, GSE58812, GSE88770 |
| Treatment responsive cohort | GSE20194, GSE20271, GSE22093, GSE22513, GSE23988, GSE25055, GSE25065, GSE37946, GSE42822, GSE66399, GSE82173 |

Supplemental Table 2. 51-genes associated with recurrence of breast cancer

| **Gene Name** | **Entrez Gene ID** |
| --- | --- |
| AURKA | 6790 |
| BIRC5 | 332 |
| BUB1 | 699 |
| C6orf155 | 79940 |
| CCNB2 | 9133 |
| CCNE2 | 9134 |
| CCT2 | 10576 |
| CCT5 | 22948 |
| CDK1 | 983 |
| CENPA | 1058 |
| CENPE | 1062 |
| CENPF | 1063 |
| CENPN | 55839 |
| CKS2 | 1164 |
| DSCC1 | 79075 |
| DTL | 51514 |
| E2F1 | 1869 |
| E2F8 | 79733 |
| FADD | 8772 |
| FOXM1 | 2305 |
| FXR1 | 8087 |
| HSPB1 | 3315 |
| JMJD6 | 23210 |
| KIAA0101 | 9768 |
| KIF11 | 3832 |
| KIF2C | 11004 |
| KPNA2 | 3838 |
| KRT23 | 25984 |
| LRRC59 | 55379 |
| MAD2L1 | 4085 |
| MCM10 | 55388 |
| MELK | 9833 |
| MKI67 | 4288 |
| MYBL2 | 4605 |
| NEK2 | 4751 |
| NME1 | 4830 |
| NUSAP1 | 51203 |
| POLQ | 10721 |
| PRC1 | 9055 |
| PSMD2 | 5708 |
| PSMD7 | 5713 |
| RACGAP1 | 29127 |
| RECQL4 | 9401 |
| RPL29 | 6159 |
| RRM2 | 6241 |
| SLC19A1 | 6573 |
| TIMM17A | 10440 |
| TOP2A | 7153 |
| TRIP13 | 9319 |
| UBE2C | 11065 |
| UBE2S | 27338 /// 246719 |

Supplemental Table S3: Kaplan–Meier recurrence free survival at 5 and 10 years for the validation cohort (N=1315), according to the expression of the classifier genes

|  | No. of patients | 5 years recurrence free survival (95% CI) | 10 years recurrence free survival (95% CI) |
| --- | --- | --- | --- |
| all patients | 1315 (100%) | 79% (78%-81%) | 67%(65%-69%) |
| low expression of classifier genes(log2(intensity) < 7.5) | 169 (13%) | 91% (88%-93%) | 82%(77%-86%) |
| intermediate expression of classifier genes(7.5 ≤ log2(intensity) ≤ 9) | 606 (46%) | 84% (82%-86%) | 71%(68%-74%) |
| high expression of classifier genes(log2(intensity) > 9) | 540 (41%) | 71% (69%-73%) | 58%(55%-61%) |

Supplemental Table S4. The association of pathological complete responses towards different treatment protocols according to the expression of classifier genes. FEC: a combination regimen of 5 fluorouracil, epirubicin, cyclophosphamide ; FAC: a combination regimen of 5 fluorouracil, doxorubicin (Adriamycin), and cyclophosphamide

|  |  | **pCR** | **RD** | **pCR% in total population** | **P-value** |
| --- | --- | --- | --- | --- | --- |
| **All** | **FEC-Taxotere-HER2** | 12 | 13 | 48% |  |
|  | high expression | 7 | 7 | 50% | 0.8625 |
|  | intermediate expression | 5 | 6 | 45% |  |
|  | low expression | 0 | 0 |  |  |
|  | **FEC-Taxol-HER2** | 27 | 61 | 31% |  |
|  | high expression | 24 | 52 | 32% | 0.9203 |
|  | intermediate expression | 2 | 9 | 18% |  |
|  | low expression | 1 | 0 | 100% |  |
|  | **FEC-Taxol** | 15 | 72 | 17% |  |
|  | high expression | 2 | 17 | 11% | 0.5777 |
|  | intermediate expression | 12 | 40 | 23% |  |
|  | low expression | 1 | 14 | 7% |  |
|  | **only FAC** | 2 | 27 | 7% |  |
|  | high expression | 0 | 11 | 0% | 0.6985 |
|  | intermediate expression | 2 | 16 | 11% |  |
|  | low expression | 0 | 0 |  |  |
|  | **Only Taxol** | 4 | 13 | 24% |  |
|  | high expression | 4 | 10 | 29% | 0.7518 |
|  | intermediate expression | 0 | 2 | 0% |  |
|  | low expression | 0 | 1 | 0 |  |
| **ER+** | **FEC-Taxotere-HER2** | 2 | 8 | 20% |  |
|  | high expression | 2 | 5 | 29% | 0.8625 |
|  | intermediate expression | 0 | 3 | 0% |  |
|  | low expression | 0 | 0 |  |  |
|  | **FEC-Taxol-HER2** | 0 | 0 |  |  |
|  | high expression | 0 | 0 |  |  |
|  | intermediate expression | 0 | 0 |  |  |
|  | low expression | 0 | 0 |  |  |
|  | **FEC-Taxol** | 3 | 50 | 6% |  |
|  | high expression | 1 | 9 | 10% | 0.9203 |
|  | intermediate expression | 2 | 28 | 7% |  |
|  | low expression | 0 | 13 | 0% |  |
|  | **only FAC** | 1 | 15 | 6% |  |
|  | high expression | 0 | 6 | 0% | 0.7913 |
|  | intermediate expression | 1 | 9 | 10% |  |
|  | low expression | 0 | 0 |  |  |
|  | **Only Taxol** | 0 | 0 |  |  |
|  | high expression | 0 | 0 |  |  |
|  | intermediate expression | 0 | 0 |  |  |
|  | low expression | 0 | 0 |  |  |
|  | **Only Hormone therapy** | 9 | 8 | 53% |  |
|  | high expression | 9 | 8 | 53% | <.0001 |
|  | intermediate expression | 0 | 0 |  |  |
|  | low expression | 0 | 0 |  |  |
| **ER-** | **FEC-Taxotere-HER2** | 10 | 5 | 67% |  |
|  | high expression | 5 | 2 | 71% | 0.8625 |
|  | intermediate expression | 5 | 3 | 63% |  |
|  | low expression | 0 | 0 |  |  |
|  | **FEC-Taxol-HER2** | 0 | 0 |  |  |
|  | high expression | 0 | 0 |  |  |
|  | intermediate expression | 0 | 0 |  |  |
|  | low expression | 0 | 0 |  |  |
|  | **FEC-Taxol** | 12 | 21 | 36% |  |
|  | high expression | 1 | 8 | 11% | 0.1502 |
|  | intermediate expression | 10 | 12 | 45% |  |
|  | low expression | 1 | 1 | 50% |  |
|  | **only FAC** | 1 | 12 | 8% |  |
|  | high expression | 0 | 5 | 0% | 0.8065 |
|  | intermediate expression | 1 | 7 | 13% |  |
|  | low expression | 0 | 0 |  |  |
|  | **Only Taxol** | 0 | 3 | 0% |  |
|  | high expression | 0 | 2 | 0% |  |
|  | intermediate expression | 0 | 1 | 0% |  |
|  | low expression | 0 | 0 |  |  |

Supplemental Table 5. The distribution of pathological complete responses in relation to different patient characteristics.

|  | **PCR** | **RD** | **PCR% in total population** | **P value** |
| --- | --- | --- | --- | --- |
| **All patients expression of classifier genes** | 334 | 1031 | 24% |  |
| High expression | 229 | 415 | 36% | <.0001 |
| Intermediate expression | 95 | 513 | 16% |  |
| Low expression | 10 | 103 | 9% |  |
| **Age <= 50** |  |  |  |  |
| All patients expression of classifier genes | 168 | 500 | 25% |  |
| High expression | 116 | 187 | 38% | <.0001 |
| Intermediate expression | 46 | 264 | 15% |  |
| Low expression | 6 | 49 | 11% |  |
| **Age <= 50** |  |  |  |  |
| All patients expression of classifier genes | 125 | 449 | 22% |  |
| High expression | 76 | 160 | 32% | 0.0031 |
| Intermediate expression | 46 | 236 | 16% |  |
| Low expression | 3 | 53 | 5% |  |
| **Tumore size <= 2cm** |  |  |  |  |
| All patients expression of classifier genes | 22 | 71 | 24% |  |
| High expression | 14 | 22 | 39% | 0.0652 |
| Intermediate expression | 7 | 34 | 17% |  |
| Low expression | 1 | 15 | 6% |  |
| **Tumore size > 2cm** |  |  |  |  |
| All patients expression of classifier genes | 221 | 823 | 21% |  |
| High expression | 150 | 308 | 33% | <.0001 |
| Intermediate expression | 64 | 431 | 13% |  |
| Low expression | 7 | 84 | 8% |  |
| **Node Postive** |  |  |  |  |
| All patients expression of classifier genes | 177 | 604 | 23% |  |
| High expression | 123 | 246 | 33% | <.0001 |
| Intermediate expression | 50 | 301 | 14% |  |
| Low expression | 4 | 57 | 7% |  |
| **Node negative** |  |  |  |  |
| All patients expression of classifier genes | 66 | 297 | 18% |  |
| High expression | 41 | 85 | 33% | 0.0355 |
| Intermediate expression | 21 | 169 | 11% |  |
| Low expression | 4 | 43 | 9% |  |
| **Well differentiated** |  |  |  |  |
| All patients expression of classifier genes | 2 | 59 | 3% |  |
| High expression | 2 | 4 | 33% | 1 |
| Intermediate expression | 0 | 28 | 0% |  |
| Low expression | 0 | 27 | 0% |  |
| **Intermediate differentiated** |  |  |  |  |
| All patients expression of classifier genes | 32 | 353 | 8% |  |
| High expression | 11 | 84 | 12% | 0.0037 |
| Intermediate expression | 19 | 218 | 8% |  |
| Low expression | 2 | 51 | 4% |  |
| **Poorly differentiated** |  |  |  |  |
| All patients expression of classifier genes | 190 | 421 | 31% |  |
| High expression | 135 | 217 | 38% | <.0001 |
| Intermediate expression | 49 | 181 | 21% |  |
| Low expression | 6 | 23 | 21% |  |
| **ER positive** |  |  |  |  |
| All patients expression of classifier genes | 92 | 605 | 13% |  |
| High expression | 71 | 192 | 27% | 0.3272 |
| Intermediate expression | 18 | 326 | 5% |  |
| Low expression | 3 | 87 | 3% |  |
| **ER negative** |  |  |  |  |
| All patients expression of classifier genes | 210 | 349 | 38% |  |
| High expression | 129 | 161 | 44% | <.0001 |
| Intermediate expression | 75 | 173 | 30% |  |
| Low expression | 6 | 15 | 29% |  |
| **TNBC positive** |  |  |  |  |
| All patients expression of classifier genes | 95 | 205 | 32% |  |
| High expression | 60 | 97 | 38% | <.0001 |
| Intermediate expression | 31 | 96 | 24% |  |
| Low expression | 4 | 12 | 25% |  |
| **TNBC negative** |  |  |  |  |
| All patients expression of classifier genes | 136 | 627 | 18% |  |
| High expression | 90 | 228 | 28% | 0.0817 |
| Intermediate expression | 40 | 312 | 11% |  |
| Low expression | 6 | 87 | 6% |  |
| **PR positive** |  |  |  |  |
| All patients expression of classifier genes | 56 | 435 | 11% |  |
| High expression | 47 | 113 | 29% | 0.1502 |
| Intermediate expression | 7 | 250 | 3% |  |
| Low expression | 2 | 72 | 3% |  |
| **PR negative** |  |  |  |  |
| All patients expression of classifier genes | 188 | 398 | 32% |  |
| High expression | 109 | 191 | 36% | <.0001 |
| Intermediate expression | 72 | 180 | 29% |  |
| Low expression | 7 | 27 | 21% |  |
| **HER2 positive** |  |  |  |  |
| All patients expression of classifier genes | 102 | 160 | 39% |  |
| High expression | 58 | 102 | 36% | 0.0003 |
| Intermediate expression | 41 | 55 | 43% |  |
| Low expression | 3 | 3 | 50% |  |
| **HER2 negative** |  |  |  |  |
| All patients expression of classifier genes | 166 | 725 | 19% |  |
| High expression | 120 | 251 | 32% | <.0001 |
| Intermediate expression | 39 | 378 | 9% |  |
| Low expression | 7 | 96 | 7% |  |
| **Invasive ductal carcinoma** |  |  |  |  |
| All patients expression of classifier genes | 78 | 329 | 19% |  |
| high expression | 41 | 122 | 25% | 0.3251 |
| intermediate expression | 33 | 168 | 16% |  |
| low expression | 4 | 39 | 9% |  |
| **Invasive lobular carcinoma** |  |  |  |  |
| All patients expression of classifier genes | 1 | 16 | 6% |  |
| high expression | 0 | 2 | 0% | 0.5837 |
| intermediate expression | 1 | 6 | 14% |  |
| low expression | 0 | 8 | 0% |  |

Supplemental Table 6: Drugs and candidate chemicals to target the 10 genes /clinical trial.gov (https://www.proteinatlas.org/)

| Gene | Selective Drug(s) approved or in trial | Ref |
| --- | --- | --- |
| ***AURKA*** | MLN8237 (Phase 1 for Patients With Advanced Solid Tumors and Metastatic Triple-negative Breast Cancer, Recruiting).  Danusertib (Phase 2 for Metastatic Hormone Refractory Prostate Cancer as inhibitor, Completed).  AT9283 (Phase 2 for Multiple Myeloma as inhibitor, Completed). | ^1-3^ |
| ***CDK1*** | P276-00 (Phase 1 for breast cancer as inhibitor, Terminated).  Terameprocol (Phase 1 for leukemia as inhibitor, Terminated).  Dinaciclib (Phase 2 for multiple myeloma as inhibitor, Recruiting).  Roscovitine (Phase 2 for anticancer as inhibitor, Withdrawn).  Alvocidib (Orphan drug designation for acute lymphocytic leukemia as inhibitor, Recruiting). | ^4-8^ |
| ***KIF11*** | ARRY-520(Phase 2 for Multiple Myeloma as inhibitor, 3 completed, 1 ongoing). | ^9^ |
| ***TOP2A*** | Etoposide (Approved for chemotherapy medication as inhibitor).  Amsacrine (Approved for acute myeloid leukaemia as inhibitor). | ^10-12^ |

1. Falchook, G. S. *et al.* Phase I/II study of weekly paclitaxel with or without MLN8237 (alisertib), an investigational aurora A kinase inhibitor, in patients with recurrent epithelial ovarian, fallopian tube, or primary peritoneal cancer (OC), or breast cancer (BrC): Phase I results. *J. Clin. Oncol.* **30**, 5021-5021 (2012).

2. Schoffski, P. *et al.* Efficacy and safety of biweekly i.v. administrations of the Aurora kinase inhibitor danusertib hydrochloride in independent cohorts of patients with advanced or metastatic breast, ovarian, colorectal, pancreatic, small-cell and non-small-cell lung cancer: a multi-tumour, multi-institutional phase II study. *Ann. Oncol.* **26**, 598-607 (2015).

3. Dent, S. F. *et al.* NCIC CTG IND.181: phase I study of AT9283 given as a weekly 24 hour infusion in advanced malignancies. *Invest. New Drugs* **31**, 1522-1529 (2013).

4. Tibes, R. *et al.* Phase I study of the novel Cdc2/CDK1 and AKT inhibitor terameprocol in patients with advanced leukemias. *Invest. New Drugs* **33**, 389-396 (2015).

5. Kumar, S. K. *et al.* Dinaciclib, a novel CDK inhibitor, demonstrates encouraging single-agent activity in patients with relapsed multiple myeloma. *Blood* **125**, 443-448 (2015).

6. Cassaday, R. D. *et al.* A phase II, single-arm, open-label, multicenter study to evaluate the efficacy and safety of P276-00, a cyclin-dependent kinase inhibitor, in patients with relapsed or refractory mantle cell lymphoma. *Clin. Lymphoma Myeloma Leuk.* **15**, 392-397 (2015).

7. Cicenas, J. *et al.* Roscovitine in cancer and other diseases. *Ann. Transl. Med.* **3**, 135 (2015).

8. Wiernik, P. H. Alvocidib (flavopiridol) for the treatment of chronic lymphocytic leukemia. *Expert Opin. Investig. Drugs* **25**, 729-734 (2016).

9. Shah, J. J. *et al.* A Phase 1 and 2 study of Filanesib alone and in combination with low-dose dexamethasone in relapsed/refractory multiple myeloma. *Cancer* **123**, 4617-4630 (2017).

10. Ketron, A. C., Denny, W. A., Graves, D. E. & Osheroff, N. Amsacrine as a topoisomerase II poison: importance of drug-DNA interactions. *Biochemistry* **51**, 1730-1739 (2012).

11. Willmore, E., Frank, A. J., Padget, K., Tilby, M. J. & Austin, C. A. Etoposide targets topoisomerase IIalpha and IIbeta in leukemic cells: isoform-specific cleavable complexes visualized and quantified in situ by a novel immunofluorescence technique. *Mol. Pharmacol.* **54**, 78-85 (1998).

12. Seymour, L. W. *et al.* Phase II studies of polymer-doxorubicin (PK1, FCE28068) in the treatment of breast, lung and colorectal cancer. *Int. J. Oncol.* **34**, 1629-1636 (2009).

Supplemental Table 7: the prediction of disease relapse by the 10 classifier genes individually among the validation cohort (N=1315 patients). P-value and hazard ratio are calculated by Cox Proportional Hazard Analysis.

| *Variable* | *Gene* | *P Value* | *Hazard Ratio (95% CI)* |
| --- | --- | --- | --- |
| *201291_s_at* | *TOP2A* | *2.00E-07* | *1.956* |
| *202705_at* | *CCNB2* | *1.00E-04* | *1.63* |
| *203214_x_at* | *CDK1* | *0.001* | *1.509* |
| *204170_s_at* | *CKS2* | *0.08* | *1.254* |
| *204444_at* | *KIF11* | *0.01* | *1.382* |
| *204947_at* | *E2F1* | *0.008* | *1.406* |
| *208079_s_at* | *AURKA* | *2.00E-04* | *1.598* |
| *211576_s_at* | *SLC19A1* | *5.00E-04* | *1.552* |
| *218009_s_at* | *PRC1* | *1.00E-05* | *1.765* |
| *219990_at* | *E2F8* | *1.00E-05* | *1.761* |

Supplemental Table 8: The prediction of response to neoadjuvant chemotherapy by the 10 classifier genes individually among the treatment responsive cohort (N= 1365 patients). P value is calculated by Chi-squares analysis.

| **Probe** | **Gene** |  | **RD** | **pCR** | **pCR% in total population** | **p-value** |
| --- | --- | --- | --- | --- | --- | --- |
| 201291_s_at | TOP2A | High expression | 484 | 228 | 32% | <.0001 |
|  |  | Low expression | 547 | 106 | 16% |  |
| 201292_at | TOP2A | High expression | 508 | 221 | 30% | <.0001 |
|  |  | Low expression | 523 | 113 | 18% |  |
| 202705_at | CCNB2 | High expression | 495 | 243 | 33% | <.0001 |
|  |  | Low expression | 536 | 91 | 15% |  |
| 203214_x_at | CDK1 | High expression | 474 | 228 | 32% | <.0001 |
|  |  | Low expression | 557 | 106 | 16% |  |
| 204170_s_at | CKS2 | High expression | 489 | 231 | 32% | <.0001 |
|  |  | Low expression | 542 | 103 | 16% |  |
| 204444_at | KIF11 | High expression | 502 | 242 | 33% | <.0001 |
|  |  | Low expression | 529 | 92 | 15% |  |
| 204947_at | E2F1 | High expression | 484 | 195 | 29% | 0.0004 |
|  |  | Low expression | 547 | 139 | 20% |  |
| 208079_s_at | AURKA | High expression | 477 | 221 | 32% | <.0001 |
|  |  | Low expression | 554 | 113 | 17% |  |
| 211576_s_at | SLC19A1 | High expression | 511 | 195 | 28% | <.0001 |
|  |  | Low expression | 520 | 139 | 21% |  |
| 218009_s_at | PRC1 | High expression | 507 | 255 | 33% | <.0001 |
|  |  | Low expression | 524 | 79 | 13% |  |
| 219990_at | E2F8 | High expression | 541 | 243 | 31% | <.0001 |
|  |  | Low expression | 490 | 91 | 16% |  |

Supplemental Table 9. Top 10 ranking differential expression genes identified by matched sample comparison of 43 paired cancer and normal tissues as detailed in Supplemental Figure 1

| Gene Symbol | Probes | P (t-test) |
| --- | --- | --- |
| CIDEC | 219398_at | 2.72E-19 |
| GYG2 | 210964_s_at | 6.82E-19 |
| PPP1R1A | 205478_at | 1.34E-18 |
| RBP4 | 219140_s_at | 1.57E-18 |
| HCAR3 | 205220_at | 6.15E-18 |
| PDE3B | 222317_at | 1.38E-17 |
| RGS1 | 202988_s_at | 6.99E-17 |
| CD24 | 266_s_at | 9.67E-17 |
| PCOLCE2 | 219295_s_at | 1.72E-16 |
| MAOA | 212741_at | 1.76E-16 |

Supplemental Table 10. Top 10 ranking disease relapse related genes selected by conventional machine learning technique as detailed in Supplemental Figure 2

| **Gene Symbol** | **Probes** | **P (t-test)** | **P (logrank)** |
| --- | --- | --- | --- |
| TMEM97 | 212281_s_at | 4.44E-10 | 6.26E-05 |
| GID8 | 218448_at | 3.75E-07 | 6.21E-05 |
| NUSAP1 | 218039_at | 7.53E-07 | 1.40E-06 |
| NEK11 | 219542_at | 9.08E-06 | 1.82E-05 |
| CX3CR1 | 205898_at | 0.000011 | 3.05E-06 |
| GNAI3 | 201180_s_at | 1.14E-05 | 4.10E-05 |
| RDX | 212398_at | 1.16E-05 | 2.56E-05 |
| DHX40 | 218277_s_at | 1.82E-05 | 1.98E-05 |
| MAP1LC3B | 208785_s_at | 2.59E-05 | 2.45E-05 |
| RANBP1 | 202483_s_at | 3.08E-05 | 1.63E-06 |

Supplemental Table 11. An evaluation of the top 10 mechanistically relevant classifier genes identified by pathway enrichment analysis, machine learning techniques, and other statistical procedures to predict response to chemotherapy.

|  |  | | The 10 mechanistically relevant classifier genes  (see Figure 1) | | | The 10 top ranking relapsed related genes defined by machine learning technique  (see supplemental Figure 3) | | | The 10 top ranking genes defined by microarray statistical analysis  (see supplemental Figure 2) | | |
| --- | --- | --- | --- | --- | --- | --- | --- | --- | --- | --- | --- |
|  |  | | pCR/RD | pCR% | P-value | pCR/RD | pCR% | P-value | pCR/RD | pCR% | P-value |
| All patients | | All patients expression of classifier genes |  |  |  |  |  |  |  |  |  |
|  |  | Overall | 334/1031 | 24% |  | 334/1031 | 24% |  | 334/1031 | 24% |  |
|  |  | High expression | 229/415 | 36% | **<0.001** | 208/436 | 32% | **<0.001** | 186/458 | 29% | **<0.001** |
|  |  | Intermediate expression | 95/513 | 16% |  | 116/492 | 19% |  | 132/476 | 22% |  |
|  |  | Low expression | 10/103 | 9% |  | 10/103 | 9% |  | 16/97 | 14% |  |
|  |  | FEC-Taxotere |  |  |  |  |  |  |  |  |  |
|  |  | Overall | 25/41 | 38% |  | 25/41 | 38% |  | 25/41 | 38% |  |
|  |  | High expression | 21/21 | 50% | **<0.05** | 18/22 | 45% | 0.2222 | 11/16 | 41% | 0.8875 |
|  |  | Intermediate expression | 4/20 | 17% |  | 7/17 | 29% |  | 13/24 | 35% |  |
|  |  | Low expression | 0/0 |  |  | 0/2 | 0% |  | 1/1 | 50% |  |
|  |  | FAC-Taxol |  |  |  |  |  |  |  |  |  |
|  |  | Overall | 83/273 | 23% |  | 83/273 | 23% |  | 83/273 | 23% |  |
|  |  | High expression | 57/107 | 35% | **<.001** | 51/107 | 32% | **<0.001** | 39/100 | 28% | 0.1175 |
|  |  | Intermediate expression | 23/134 | 15% |  | 28/141 | 17% |  | 39/139 | 22% |  |
|  |  | Low expression | 3/32 | 9% |  | 4/25 | 14% |  | 5/34 | 13% |  |
|  |  | Only FEC |  |  |  |  |  |  |  |  |  |
|  |  | Overall | 30/104 | 22% |  | 30/104 | 22% |  | 30/104 | 22% |  |
|  |  | High expression | 18/31 | 37% | **<0.01** | 11/45 | 20% | 0.6629 | 16/46 | 26% | 0.5023 |
|  |  | Intermediate expression | 12/63 | 16% |  | 18/48 | 27% |  | 11/47 | 19% |  |
|  |  | Low expression | 0/10 | 0% |  | 1/11 | 8% |  | 3/11 | 21% |  |
| ER+ | | All patients expression of classifier genes |  |  |  |  |  |  |  |  |  |
|  |  | Overall | 92/605 | 13% |  | 92/605 | 13% |  | 92/605 | 13% |  |
|  |  | High expression | 71/192 | 27% | **<0.001** | 53/208 | 20% | **<0.001** | 44/202 | 18% | **<0.01** |
|  |  | Intermediate expression | 18/326 | 5% |  | 37/320 | 10% |  | 41/319 | 11% |  |
|  |  | Low expression | 3/87 | 3% |  | 2/77 | 3% |  | 7/84 | 8% |  |
|  |  | FEC-Taxotere |  |  |  |  |  |  |  |  |  |
|  |  | Overall | 8/20 | 29% |  | 8/20 | 29% |  | 8/20 | 29% |  |
|  |  | High expression | 6/7 | 46% | 0.1345 | 6/10 | 38% | 0.431 | 5/5 | 50% | 0.1512 |
|  |  | Intermediate expression | 2/13 | 13% |  | 2/8 | 20% |  | 3/14 | 18% |  |
|  |  | Low expression | 0/0 |  |  | 0/2 | 0% |  | 0/1 | 0% |  |
|  |  | FAC-Taxol |  |  |  |  |  |  |  |  |  |
|  |  | Overall | 20/185 | 10% |  | 20/185 | 10% |  | 20/185 | 10% |  |
|  |  | High expression | 18/65 | 22% | **<0.001** | 11/63 | 15% | 0.1082 | 9/50 | 15% | 0.1532 |
|  |  | Intermediate expression | 1/93 | 1% |  | 8/102 | 7% |  | 8/104 | 7% |  |
|  |  | Low expression | 1/27 | 4% |  | 1/20 | 5% |  | 3/31 | 9% |  |
|  |  | Only FEC |  |  |  |  |  |  |  |  |  |
|  |  | Overall | 10/49 | 17% |  | 10/49 | 17% |  | 10/49 | 17% |  |
|  |  | High expression | 5/15 | 25% | 0.4166 | 4/23 | 15% | 1 | 4/14 | 22% | 0.7401 |
|  |  | Intermediate expression | 5/28 | 15% |  | 6/20 | 23% | 0.431 | 6/26 | 19% |  |
|  |  | Low expression | 0/6 | 0% |  | 0/6 | 0% |  | 0/9 | 0% |  |
| ER- | | All patients expression of classifier genes |  |  |  |  |  |  |  |  |  |
|  |  | Overall | 210/349 | 38% |  | 210/349 | 38% |  | 210/349 | 38% |  |
|  |  | High expression | 129/161 | 44% | **<0.001** | 124/160 | 44% | **<0.01** | 111/189 | 37% | 0.8415 |
|  |  | Intermediate expression | 75/173 | 30% |  | 78/166 | 32% |  | 90/147 | 38% |  |
|  |  | Low expression | 6/15 | 29% |  | 8/23 | 26% |  | 9/13 | 41% |  |
|  |  | FEC-Taxotere |  |  |  |  |  |  |  |  |  |
|  |  | Overall | 17/20 | 46% |  | 17/20 | 46% |  | 17/20 | 46% |  |
|  |  | High expression | 15/14 | 52% | **<0.05** | 12/12 | 50% | 0.7401 | 6/10 | 38% | 0.5716 |
|  |  | Intermediate expression | 2/6 | 25% |  | 5/8 | 38% |  | 10/10 | 50% |  |
|  |  | Low expression | 0/0 |  |  | 0/0 |  |  | 1/0 | 100% |  |
|  |  | FAC-Taxol |  |  |  |  |  |  |  |  |  |
|  |  | Overall | 63/88 | 42% |  | 63/88 | 42% |  | 63/88 | 42% |  |
|  |  | High expression | 39/42 | 48% | 0.1198 | 40/44 | 48% | 0.1389 | 30/50 | 38% | 0.3401 |
|  |  | Intermediate expression | 22/41 | 35% |  | 20/39 | 34% |  | 31/35 | 47% |  |
|  |  | Low expression | 2/5 | 29% |  | 3/5 | 38% |  | 2/3 | 40% |  |
|  |  | Only FEC |  |  |  |  |  |  |  |  |  |
|  |  | Overall | 20/55 | 27% |  | 20/55 | 27% |  | 20/55 | 27% |  |
|  |  | High expression | 13/16 | 45% | **<0.01** | 7/22 | 24% | 0.8875 | 12/32 | 27% | 0.8860 |
|  |  | Intermediate expression | 7/35 | 17% |  | 12/28 | 30% |  | 5/21 | 19% |  |
|  |  | Low expression | 0/4 | 0% |  | 1/5 | 17% |  | 3/2 | 60% |  |
